# Trends in utilization of stored cryopreserved autologous peripheral hematopoietic cells intended for a second (or beyond) autologous hematopoietic cell transplantation in patients with multiple myeloma: a single center experience

**DOI:** 10.1038/s41409-023-02035-y

**Published:** 2023-07-21

**Authors:** Farah Yassine, Mohamed A. Kharfan-Dabaja, Athanasios Tsalantsanis, Vivek Roy, Abba C. Zubair, Hemant S. Murthy, Ernesto Ayala, Madiha Iqbal, Taimur Sher, Sikander Ailawadhi, Ricardo D. Parrondo

**Affiliations:** 1 Division of Hematology-Oncology and Blood and Marrow Transplantation Program, Mayo Clinic, Jacksonville, FL USA; 2 Program for Comparative Effectiveness Research, Morsani College of Medicine, University of South Florida, Tampa, FL USA; 3 Department of Pathology and Laboratory Medicine, Mayo Clinic, Jacksonville, FL USA

**Keywords:** Stem-cell research, Myeloma

## Abstract

Due to the advent of effective novel therapies for multiple myeloma (MM), the use of cryopreserved autologous peripheral blood hematopoietic cells (APBHC) for a salvage autologous transplant (auto-HCT) is in decline. We evaluated utilization trends and costs associated with cryopreserved APBHC in patients with MM. We retrospectively evaluated the clinicopathologic data from 440 patients with MM who underwent APBHC mobilization and collection at Mayo Clinic Florida between 2010 and 2019. Based on institution-specific charges as of May 2021, the cost of 1 session of APBHC collection/apheresis was $4,680 and the cost of 1 year of APBHC cryopreservation was $4,790 per patient. Out of 347 patients who had APBHC in cryopreservation, 5 (1.4%) underwent a salvage auto-HCT and 61% of patients had ≥1 excess collection sessions for APBHC that ultimately went unused. The median cost of excess collection sessions was $4,680 per patient (range, $4,680-$32,760) and the median total cost for excess collection sessions plus costs for storage was $23,840 per patient (range, $4,680–$85,450). The sum of costs of excess collection sessions was $2,077,920 and the sum of costs of cryopreservation was $5,812,665. Institutional policies regarding universal APBHC collection and long-term storage should be reevaluated in the era of novel therapeutics.

## Introduction

The standard of care treatment for transplant-eligible patients with multiple myeloma (MM) is high dose melphalan followed by autologous hematopoietic stem cell transplantation (auto-HCT) consolidation [1–3], usually preceded by an induction regimen consisting of a proteasome inhibitor (PI), an immunomodulatory agent (IMiD) and dexamethasone [4–6]. The advent of novel agents, including next generation PIs, monoclonal antibodies (MoAb), chimeric antigen receptor T-cell (CAR T) therapy, and beyond, has markedly improved the survival of patients with MM [7–10]. For instance, after a median follow-up of 67 months, patients treated with bortezomib, lenalidomide and dexamethasone (VRD) induction, followed by auto-HCT, achieved an overall response rate (ORR) of 98.5%, an estimated progression-free survival (PFS) of 65 months, and a median overall survival (OS) of 126.6 months [11]. However, auto-HCT is not a curative option, and all patients are expected to relapse eventually [12, 13]. A salvage or second auto-HCT has historically been an effective approach for relapsed MM depending on the patient’s functional status, disease characteristics, as well as response to first transplant and previous lines of therapy [14–16]. The recent aforementioned advances in novel therapies have significantly improved the median OS rates of patients with MM [11], challenging the role of salvage auto-HCT for relapsed disease. Phipps and colleagues reported a decline in the trend of performing a second transplant, with only 19% of patients with MM receiving a second auto-HCT between 1993 and 2011 at the Fred Hutchinson Cancer Research Center [17]. Nonetheless, the national Comprehensive cancer Network (NCCN) panel still recommends harvesting autologous peripheral blood hematopoietic cells (APBHC) enough for performing at least two transplants at the time of the first auto-HCT [18]. Recent studies have shown that with each decade after 1990, the use of cryopreserved APBHC for a salvage auto-HCT has declined and is now in the range of 4.6–15% in the decade between 2010–2018 [17]. Therefore, while the common practice in most centers is to perform extra apheresis and collection sessions, the declining utilization trend is incurring additional costs of APBHC harvest and storage. Phipps et al. reported an average extra cost of $4,981.12 per patient between 1993 and 2011 [17], and along the same lines, Chhabra et al. reported an estimated $9,336 per patient spent on excess collection, cryopreservation, and storage between 2012 and 2017 [19].

The revolution of targeted therapies for MM in the 2010–2020 decade continues to introduce highly efficacious and well-tolerated targeted and immunotherapies for relapsed/refractory myeloma [20], limiting the role of salvage auto-HCT. However, there is limited data on the actual use of the excess APBHC in a second auto-HCT in the 2010 decade and whether extra stem cells should be harvested in all or a specific patient population with MM. Considering the rapidly evolving treatment landscape of MM, we sought to evaluate the trends and associated costs of collection, storage, and utilization of APBHC intended for a second (or beyond) auto-HCT in patients with MM and highlight the need to re-evaluate the prevailing practice guidelines for optimal use of healthcare resources.

## Methods

### Subjects

We conducted a retrospective single center study using the clinical and laboratory databases at the Mayo Clinic in Florida (MCF). The study was approved by the Institutional Review Board at MCF (IRB# 20-006432). A total of 440 adult patients (age ≥18 years) with a confirmed diagnosis of MM who underwent APBHC mobilization and collection between 2010 and 2019 were included. We extracted demographical patient information as well as clinical data including disease characteristics, previous lines of therapies, response, progression, relapse, number of mobilization and apheresis sessions, and number of APBHC collected, used, and stored. APBHC mobilization included any method such as granulocyte-colony stimulating factor (G-CSF) alone or with plerixafor.

### Cost of collection and storage

The target APBHC cell count for one auto-HCT at our center is 3 × 10^6^ CD34^+^ cells per Kg. We estimated the costs involved in APBHC collection (apheresis) and cryopreservation storage based on our institution-specific charges as of May 2021. The cost of 1 session of APBHC collection/apheresis was $4,680 and the cost of 1 year of APBHC cryopreservation was $4790 per patient.

### Statistical analysis

Statistical analysis was performed using JMP Pro 15 (SAS). Descriptive statistics were performed on the variables of interest. We report frequencies and percentages as well as medians and ranges where appropriate. Survival analysis on all patients who underwent at least 1 auto-HCT was carried out using the Kaplan–Meier method. We report the mean and 95% confidence intervals (95% CI) for overall survival (OS) and progression free survival (PFS).

## Results

### Patient demographics

Patient demographics are summarized in Table 1. A total of 440 patients who underwent at least one APBHC collection session between 2010 and 2019 at MCF were included in the analysis. Out of this total, 347 patients underwent at least one auto-HCT. The median age of included patients at the time of stem cell collection was 61 years (range, 28 to 75 years), and 54.8% (*n* = 241) of the patients were male. The majority of auto-HCT were upfront (88.2%, *n* = 306) while only 11.8% (*n* = 41) occurred at first disease relapse. The number of regimens received by patients prior to auto-HCT ranged between 1 and 13, with a median of 1 line of induction therapy. The triplet induction regimen VRD was the most common (44.6%, *n* = 155), while other induction regimen included bortezomib-cyclophosphamide-dexamethasone (VCD) (20.7%, *n* = 72), VD (12.4%, *n* = 43) and RD (12.1%, *n* = 42). Using the MM International Staging System (ISS) for prognostication, most patients were deemed low risk within stage 1 (31.1%, *n* = 108), while 17.3% (*n* = 60) were stage II and 29.7% (*n* = 103) were stage III. However, using the revised ISS (R-ISS) scoring, the majority of patients (40.1%, *n* = 139) were in stage II, with 18.4% (*n* = 64) in stage I and 10.7% (*n* = 37) in stage III. Most of the included patients (52.7%, *n* = 183) did not have high risk cytogenetic features on Fluorescence In Situ Hybridization (FISH) testing.

**Table 1:** Patient demographics (*N* = 440).

Characteristic	Value
Male, *n* (%)	241 (54.8)
Age at APBHC Collection, median, years (range)	61 (28–75)
Underwent at least 1 auto-HCT, *n* (%)	347 (78.9)
Transplant Timing, *n* (%)	
Upfront:	306 (88.2)
At First Relapse:	41(11.8)
Number of regimens prior to auto-HCT, median (range)	1.0 (1–13)
Induction Regimens, *n* (%)	
RD	42 (12.1)
VD	43 (12.4)
VCD	72 (20.7)
VRD	155 (44.6)
Other	35 (10.2)
ISS Stage, *n* (%)	
I	108 (31.1)
II	60 (17.3)
III	103 (29.7)
Unknown	76 (21.9)
R-ISS Stage, *n* (%)	
I	64 (18.4)
II	139 (40.1)
III	37 (10.7)
Unknown	107 (30.8)
High Risk FISH, *n* (%)	
Yes	100 (28.8)
No	183 (52.7)
Unknown	64 (18.4)

*APBHC* autologous peripheral blood hematopoietic cells, *auto-HCT* autologous hematopoietic cell transplantation, *RD* lenalidomide-dexamethasone, *VD* bortezomib-dexamethasone, *VCD* bortezomib-cyclophosphamide-dexamethasone, *VRD* bortezomib-lenalidomide-dexamethasone, *ISS* international staging system, *R-ISS* revised ISS, *FISH* Fluorescence In Situ Hybridization.

### Characteristics of APBHC collection

The median number of APBHC collected was 7.6 × 10^6^ (range: 0.07 × 10^6^– 22.9 × 10^6^), and the median number of infused cells was 4 × 10^6^ (range: 1.96 × 10^6^–8.78 × 10^6^) (Table 2). The median number of mobilization sessions was 1(range: 1.0-1.03). Out of the 440 patients with MM included in the analysis, 371 patients (84.3%) required more than one collection/apheresis sessions. The median number of collection/apheresis sessions performed was 2 (range: 1–7).

**Table 2:** Characteristics of APBHC mobilization and collection/apheresis in patients with MM undergoing auto-HCT.

Characteristic	Value
Number of APBHC collected, x10^6^, median (range)	7.6 (0.07– 22.9)
Number of infused cells, x10^6^, median (range)	4.0 (1.96–8.78)
Number of mobilization attempts, median (range)	1.0 (1–2)
Number of collection/apheresis sessions, median (range)	2.0 (1–7)
Patients requiring one or more collection/apheresis session, *n* (%)	
One	68.0 (15.5%)
More than one	371.0 (84.3%)

*APBHC* autologous peripheral blood hematopoietic cells, *MM* multiple myeloma, *auto-HCT* autologous hematopoietic cell transplantation.

### Utilization and cryopreservation of collected APBHCs and associated costs

The median cost of the total collection sessions was defined as the number of apheresis/collection session multiplied by the cost of one apheresis/collection session ($4,680.0), yielding a total of $9,360.0 per patient (range: $4,680.0–$32,760.0), which is $936.0 per patient per year (Table 3). As for cell storage, the median cost of APBHC cryopreservation, defined as the number of years of storage multiplied by the cost of storage per year ($4,790.0), totaled $19,160.0 per patient (range: 0–$52,690.0), which is an average of $1,916.0 per patient per year. The median number of years of storage of APBHC that were collected but not infused in auto-HCT was 4 years (range:0–11.0). Seventy-seven (17.5%) patients had APBHC in storage for less than 2 years, while 218 (49.5%) had APBHC in storage for 2–5 years, and 145 (33%) of the patients had APBHC in storage for longer than 5 years. Of note, the median time from MM diagnosis until infusion of auto-HCT was 9.0 months (range: 0–197). Based on institutional guidelines at MCF, at least 3 × 10^6^ CD34^+^ cells/kg are infused per one auto-HCT. Most patients (82.5%, *n* = 363) collected enough APBHCs for two or more auto-HCTs (that is ≥6 × 10^6^ CD34^+^ cells/kg). During a first auto-HCT, only 8.4% (*n* = 29) of patients had all their APBHCs infused, while the majority of 83.5% (*n* = 288) had half of their collected APBHCs infused, and 8.1% (*n* = 28) had another fraction of APBHCs infused, resulting in around 91.6% (*n* = 316) of patients having a remaining fraction of collected APBHCs subject to cryopreservation and storage for potential future use. Out of the 316 patients who had extra cryopreserved APBHCs in storage, only 1.4% (*n* = 5) underwent a salvage auto-HCT and 0.9% (*n* = 3) a tandem auto-HCT. As such, the majority of patients (70%) had APBHC in storage that ultimately went unused. The median cost of unnecessary excess APBHC collection sessions was $4,680 per patient, with a range between $4,680 and $32,760. The median total cost of excess collection sessions combined with the cost of cryopreservation and storage of unused APBHC was $23,840 per patient, ranging between $4,680 and $85,450, which corresponds to $2,384 per patient per year. The sum of costs of excess collection sessions for all included patients was $2,077,920 and the sum of costs of cryopreservation for the cohort was $5,812,665.

**Table 3:** Utilization and cryopreservation of collected APBHCs and associated costs.

Characteristic	Value
Cost of apheresis/collection sessions per patient, median (range)	$9360.0 ($4,680.0–$32,760.0)
Cost of APBHC cryopreservation per patient, median (range)	$19,160.0 (0–$52,690.0)
Years of storage,	
All, median (range)	4 (0–11)
<2 years, *n* (%)	77 (17.5%)
2–5 years, *n* (%)	218 (49.5%)
>5 years, *n* (%)	145 (33.0%)
Time from MM diagnosis to auto-HCT, months, median (range)	9.0 (0–197.0)
Patients collecting enough for ≥2 auto-HCT, *n* (%)	363 (82.5%)
Number of patients with respective APBHC infused at first auto-HCT, *n* (%)	
All APBHC	29 (8.4%)
Half APHC	288 (83%)
Another fraction of APBHC	28 (8.1%)
Patients who underwent a second auto-HCT, *n* (%)	
Salvage	5 (1.4%)
Tandem	3 (0.9%)
Cost of excess APBHC collection/apheresis per patient, median (range)	$4,680.0 ($4,680.0–$32,760.0)
Cost of excess APBHC collection/apheresis sessions+cost of cryopreservation and storage per patient, median (range)	$23,840.0 ($4,680.0–$85,450.0)
Total cost of excess APBHC collection sessions for all patients	$2,077,920.0
Total cost of cryopreservation of excess APBHC for all patients	$5,812,665.0

*APBHC* autologous peripheral blood hematopoietic cells, *MM* multiple myeloma, *auto-HCT* autologous hematopoietic cell transplantation.

### Survival outcomes after the First auto-HCT

The mean PFS of the entire cohort after the first auto-HCT was 50 months (95% CI: 44.8–55.2). The mean OS for the entire cohort after the first auto-HCT was 94.8 months (95% CI: 88.6–100.9)

## Discussion

Our analysis evaluated the utilization of stored cryopreserved APBHC for a salvage auto-HCT in patients with MM. We showed that a second auto-HCT was only performed in less than 3% of the included cohort, and that extra collections and prolonged cryopreservation of APBHC incurred an extra total cost of around $7.9 million. Our data support the fact that the current practice of collecting and cryopreserving excess APBHC for potential future salvage transplants incurs extra unnecessary costs and warrants reconsideration.

At our center, the minimum number of APBHC required for an auto-HCT is 2 × 10^6^ CD34+ cells/kg while 3 to 5 × 10^6^ CD34+ cells/kg are considered acceptable and in order to preserve the option of performing a second or third salvage auto-HCT, an attempt to collect for at least two or more ASCTs in most patients aged under 70 years is recommended [21]. Multiple efforts were undertaken to determine the optimal dose of APBHC for auto-HCT. The ideal dose ranges between 3 and 5 million APBHC per Kg of patient’s weight, and studies have described an optimal dose of 8 million cells/Kg [22]. An average of 2.5 million cells/kg was described as the minimum required dose for an APBHC, and threshold below which a delayed engraftment and slower hematopoietic recovery is suffered [22, 23]. Infusion of higher stem cell doses,10–15 million cells/Kg, did not confer a difference in engraftment time or symptom burden after auto-HCT, compared to standard doses of 4–6 million cells/Kg [24]. Yet, many transplant centers follow the common practice of collecting an average of 6 to 10 million APBHC/Kg that would be enough for two auto-HCTs [25], and most patients still undergo repeated harvesting to achieve this goal [17]. Growing evidence supports the declining trend of utilization of stored stem cells for a second auto-HCT. In a single-center analysis, Chhabra et al. estimated the utilization of stored APBHC for salvage auto-HCT to be 0.8% at 2 years, and 12% at a 6-year follow-up [19]. Similarly, Wolf et al. showed a declining utilization trend in an analysis of multicenter data from the UK National Health Service Blood and Transplant (NHSBT) laboratories whereby a second transplantation was only performed in 567 patients (20%) out of 2769 who had ongoing storage of cells after a first auto-HCT in a cohort of 4026 patients with MM [26]. The declining trend of APBHC use was a recurrent theme in another single-center analysis by Liang et al., where only 18 (4.6%) out of 393 patients used backup stem cells for a second salvage auto-HCT, while 10 (2.3%) patients needed excess APBHC for stem cell boosts [27]. Similar to our study, the aforementioned studies highlight the significant extra cost involved in APBHC storage and cryopreservation. In their cost analysis, Chhabra et al. estimated a residual extra cost of $9,336 per patient, equivalent to a total of $3.73 M for the cohort [19]. As per Wolf et al., APBHC storage for a possible second transplant had incurred an additional cost of £2,461 per patient, adding up to a total cost of around £6.8 million [26].

The recent decades have witnessed the addition of multitude of next-generation novel therapies into the treatment armamentarium of MM. Next-generation PI, IMiD and MoAbs are being increasingly incorporated into induction, consolidation and maintenance regimens to deepen the hematologic responses and survival benefits conferred by auto-HCT, even in patients with high-risk features [28–31]. These novel therapies also tend to be better tolerated and more easily administered compared to a salvage auto-HCT where heavily pre-treated patients may have developed comorbidities and MM-related organ damage which makes them ineligible for high dose melphalan and a salvage auto-HCT. Most institutions store excess APBHC for as long as a patient is alive, and then for 6 months after a patient’s death. As such, it is inevitable that the cryopreservation cost of excess APBHC will keep increasing and considering the improved survival outcomes conferred by the advent of new cellular and immunotherapies for patients with MM [32, 33], the cost effectiveness of collecting for two auto-HCT comes into question given the decreasing utilization of these cells over time.

Another practice that is still driving excess APBHC collection is the use of tandem transplants. An integrated analysis of patient-level data from several European cooperative group studies which prospectively compared bortezomib-based vs non-bortezomib-based induction regimens before auto-HCT for newly diagnosed MM and also prospectively assigned patients to receive either a single or double auto-HCT revealed that in patients with high-risk cytogenetics [t(4;14) and/or del17p] who had not achieved a complete response after bortezomib-based induction therapy, tandem auto-HCT conferred PFS and OS advantages compared with single auto-HCT [34]. In the EMN02/HO95 trial, patients received four cycles of bortezomib-cyclophosphamide-dexamethasone (CyBorD) induction therapy followed by random assignment to either bortezomib-melphalan-prednisone (VMP) intensification or single or tandem auto-HCT. On an intention-to-treat basis, 3-year PFS was 73% for tandem ASCT versus 64% for single ASCT (*P* = 0.040). The risk of progression was reduced in patients with high-risk cytogenetics who underwent tandem auto-HCT (*P* = 0.014) and 3-year OS was also prolonged in patients who underwent tandem auto-HCT compared with single auto-HCT (89% v 82%, *P* = 0.011). While these European studies suggest a PFS and OS benefit for patients with high-risk cytogenetics who undergo auto-HCT, the multicenter (in the USA only) phase III STAMINA trial, where 55% of patients received bortezomib-lenalidomide-dexamethasone (VRD) induction and were then randomized to either single auto-HCT, tandem auto-HCT or single auto-HCT followed by VRD consolidation, did not show any survival benefit of tandem auto-HCT over single auto-HCT, even in patients with high risk cytogenetics [35]. These differences in survival benefit for high-risk patients who undergo tandem-HCT between European and American studies may be possibly due to the fact that VRD induction is used more frequently in the United States, and it is plausible that the use of two novel agents—proteasome inhibitor and immunomodulatory agent—in induction therapy abrogates the need for a tandem auto-HCT [28]. However, with longer follow-up in the STAMINA trial, 6 yr PFS in high-risk patients was 43.6% and 26% for tandem auto-HCT vs. single Auto-HCT, respectively (*p* = 0.03) [36]. An analysis of 488 patients with newly diagnosed MM and extramedullary disease from the European Society for Blood and Marrow Transplantation (EBMT) registry found a survival benefit in multivariate analysis for patients with high-risk cytogenetics who underwent tandem auto-HCT vs. single auto-HCT (hazard ratios 0.46 versus 0.64, *P* = 0.03) [37]. Differences in induction regimens used to treat MM across the world may account for the differences seen in the survival benefit afforded by tandem auto-HCT, but with the unprecedented responses rates seen with the advent of quadruplet induction regimens containing VRD plus an anti-CD38 monoclonal antibody and the use of cellular and immunotherapies such as CAR-T and bispecific antibodies in earlier lines of therapy on the horizon, the role of tandem auto-HCT appears to be fading away. These studies will help guide APBHC collections practices and applications of second/tandem auto-HCTs.

In addition, there is a known increased risk of developing second primary malignancies (SPM), including therapy-related myelodysplastic syndromes (tMDS), acute myeloid leukemia (tAML), and acute lymphoblastic leukemias (tALL) in patients with MM who undergo an initial auto-HCT followed by lenalidomide maintenance [38–41]. Furthermore, recent data from the EBMT reporting on the incidence of SPM in 130 patients with relapsed MM who undergo a salvage auto-HCT with cells procured after previous auto-HCT reported a cumulative incidence of tMDS/t-AML of 1% (95% CI, 0–3%), 3% (95% CI, 1–5%) and 4% (95% CI, 1–7%) at 2, 4 and 6 years, respectively. The cumulative incidence of other SPMs was 1% (95% CI, 0–2%), 3% (95% CI, 1–5%) and 3% (95% CI, 1–5), respectively [42]. As such, one should consider the risk of SPM when considering repeated exposure to high-dose melphalan in the setting of second auto-HCT, a risk which has not yet been shown with novel immunotherapies.

Prior to the era of novel therapies, a salvage auto-HCT afforded relapsed MM patients PFS and OS advantages in several randomized trials [43–48]. The PFS and OS benefit of a salvage auto-HCT in the era of novel immunotherapies and next generation drugs remains unknown but several trials are underway evaluating this question [49]. Excess APBHC have been utilized to perform stem cell boosts, in which the APBHC are administered without additional conditioning regimen, in case of delayed engraftment or poor graft function, to curb high-risk post-transplant complications such as bleeding and infections [50, 51]. This practice poses another challenge to reassessing institutional policies on APBHC collection and long-term storage. It is true that rescuing poor grafts with stem cell boosts is a well-established practice following allogeneic HCT (allo-HCT) [52, 53]; however, the benefits of stem cell boosts after auto-HCT, particularly for patients with MM, are not well studied and lack consensus guidelines. In a single center analysis evaluating the use of back-up stem cells collected from patients with MM, Liang et al. reported a low rate of stem cell boosts for delayed or non-engraftment, noted in only 10 (2.3%) of the cohort of 393 patients [27]. They also found that the predictors of the use of stem cell boost were a lower infused dose of APBHC at the auto-HCT and older patient age [27]. Similarly, Chhabra et al. reported a low rate of stem cell boost in 8 (2%) of the 400 patients in the cohort [19]. On the other hand, with the recent introduction of CAR-T therapy into the realm of MM treatment and the possibility of prolonged myelosuppression after CAR-T, emerging data is starting show the utility of stem cell boosts for this patient population [54, 55]. Therefore, it is possible that the utilization of stored APBHC may increase over time for possible use in the post-CAR-T setting [55]. This consideration adds another layer of challenge to modifying the current institutional guidelines and common practice of collecting and storing excess APBHC.

Our study has several limitations including its retrospective design encompassing one center only, which limits the generalizability of our conclusions. Our center has specific costs which may differ between other transplantation centers. Moreover, considering the continuous inflation, a current cost-analysis might reflect higher values, when prices are adjusted to the current inflated values, compared to the cost used for calculation during the performed analysis. Our center also follows specific guidelines and protocols pertaining to APBHC mobilization, collection and infusion. As such, a lower cost might be noted in centers that use a threshold less than 3 × 10^6^ CD34^+^ cells per Kg for auto-HCT, also necessitating a lower number of mobilization/collection sessions. Multiple studies are underway to determine the necessity of excess APBHC collection and to optimize stem cell mobilization methods with agents that decrease the needed resources (NCT 03932864; NCT04552743) [56, 57]. Given the faced-paced research realm, it is likely that other centers in the US and Europe have modified their practice guidelines regarding excess APBHC collection and salvage auto-HCT.

In conclusion, our study demonstrates that in patients with MM, excess APBHC collected at the time of the first auto-HCT are most often not utilized for a salvage transplant. This ongoing practice is incurring extra costs borne by institutions. The improved survival rates offered by the rapidly evolving new cellular and immunotherapies, as well as the declining trend of performing salvage auto-HCT warrant reconsideration of APBHC collection goals and guidelines by MM transplantation centers and working groups.

## Data Availability

The data that support the findings of this study are available on request from the corresponding author. The data are not publicly available due to privacy or ethical restrictions.
